# Supplementary material for: Predicting dietary management intention of patients with chronic kidney disease using protection motivation theory
Source: PLoS One. 2025 Mar 18;20(3):e0320340. doi: 10.1371/journal.pone.0320340 (PMC11957771; doi:10.1371/journal.pone.0320340)
Supplement: S1 Appendix — (DOCX) [file pone.0320340.s001.docx]

S1 STROBE Statement—Checklist of items that should be included in reports of cross-sectional studies

|  | Item No | Recommendation | Page No# |
| --- | --- | --- | --- |
| **Title and abstract** | 1 | (a) Indicate the study’s design with a commonly used term in the title or the abstract | 1 |
|  |  | (b) Provide in the abstract an informative and balanced summary of what was done and what was found | 1 |
| Introduction | | | |
| **Background/****rationale** | 2 | Explain the scientific background and rationale for the investigation being reported | 2-6 |
| **Objectives** | 3 | State specific objectives, including any prespecified hypotheses | 5-6 |
| Methods | | | |
| **Study design** | 4 | Present key elements of study design early in the paper | 6-8 |
| **Setting** | 5 | Describe the setting, locations, and relevant dates, including periods of recruitment, exposure, follow-up, and data collection | 6-7 |
| **Participants** | 6 | (a) Give the eligibility criteria, and the sources and methods of selection of participants | 6-7 |
| **Variables** | 7 | Clearly define all outcomes, exposures, predictors, potential confounders, and effect modifiers. Give diagnostic criteria, if applicable | 6-8 |
| **Data sources/** **measurement** | 8* | For each variable of interest, give sources of data and details of methods of assessment (measurement). Describe comparability of assessment methods if there is more than one group | 7-8 |
| **Bias** | 9 | Describe any efforts to address potential sources of bias | 6-7 |
| **Study size** | 10 | Explain how the study size was arrived at | 6-7 |
| **Quantitative** **variables** | 11 | Explain how quantitative variables were handled in the analyses. If applicable, describe which groupings were chosen and why | 7-9 |
| **Statistical** **methods** | 12 | (a) Describe all statistical methods, including those used to control for confounding | 8,9 |
|  |  | (b) Describe any methods used to examine subgroups and interactions | N.A. |
|  |  | (c) Explain how missing data were addressed | N.A.(No missing data) |
|  |  | (d) If applicable, describe analytical methods taking account of sampling strategy | N.A. |
|  |  | (e) Describe any sensitivity analyses | N.A. |
| Results | | | |
| **Participants** | 13* | (a) Report numbers of individuals at each stage of study—eg numbers potentially eligible, examined for eligibility, confirmed eligible, included in the study, completing follow-up, and analysed | 6-7 |
|  |  | (b) Give reasons for non-participation at each stage | 6-7 |
|  |  | (c) Consider use of a flow diagram | N.A. |
| **Descriptive** **data** | 14* | (a) Give characteristics of study participants (eg demographic, clinical, social) and information on exposures and potential confounders | 9,table1 |
|  |  | (b) Indicate number of participants with missing data for each variable of interest | No missing data |
| **Outcome data** | 15* | Report numbers of outcome events or summary measures | Table 2 |
| **Main results** | 16 | (a) Give unadjusted estimates and, if applicable, confounder-adjusted estimates and their precision (eg, 95% confidence interval). Make clear which confounders were adjusted for and why they were included | Table 4 |
|  |  | (b) Report category boundaries when continuous variables were categorized | N.A. |
|  |  | (c) If relevant, consider translating estimates of relative risk into absolute risk for a meaningful time period | N.A. |
| **Other analyses** | 17 | Report other analyses done—eg analyses of subgroups and interactions, and sensitivity analyses | 9-11 |
| Discussion | | | |
| **Key results** | 18 | Summarise key results with reference to study objectives | 11-14 |
| **Limitations** | 19 | Discuss limitations of the study, taking into account sources of potential bias or imprecision. Discuss both direction and magnitude of any potential bias | 15 |
| **Interpretation** | 20 | Give a cautious overall interpretation of results considering objectives, limitations, multiplicity of analyses, results from similar studies, and other relevant evidence | 15-16 |
| **Generalisability** | 21 | Discuss the generalisability (external validity) of the study results | 15-16 |
| Other information | | | |
| **Funding** | 22 | Give the source of funding and the role of the funders for the present study and, if applicable, for the original study on which the present article is based | 17 |

# Page No. refers to the number in the submitted version of the manuscript
