# Supplementary material for: Predicting dietary management intention of patients with chronic kidney disease using protection motivation theory
Source: PLoS One. 2025 Mar 18;20(3):e0320340. doi: 10.1371/journal.pone.0320340 (PMC11957771; doi:10.1371/journal.pone.0320340)
Supplement: S3 Appendix — (DOCX) [file pone.0320340.s003.docx]

**Chronic Kidney Disease Self-Management Behavior and Motivation Questionnaire**

**Dear Patient,**

**We are a health communication research team from Longhua Hospital affiliated with Shanghai University of Traditional Chinese Medicine, in collaboration with Shanghai Jiao Tong University. We are conducting a survey on the self-management behaviors, motivation, and cognition of chronic kidney disease (CKD) patients. The purpose is to provide reference for disease-related considerations and health communication strategies for CKD patients. All collected information will be anonymized and kept confidential, and will only be used for public health and health communication research. Responses to this survey will not affect your treatment. Please give as honest responses as you can. We sincerely appreciate your cooperation!**

**Part 1: Basic Information**

1. **ID (Fill-in-the-blank)________**
2. **Date of Birth (Fill-in-the-blank) ________**
3. **Gender (Single-choice)**

**○ Male**

**○ Female**

1. **Please provide the following information (Fill-in-the-blank)**
   - **Your height (in centimeters, cm) ________**
   - **Your weight (in kilograms, kg) ________**
   - **How long have you been diagnosed with chronic kidney disease? ________ (months/years)**
   - **What stage of chronic kidney disease are you currently in? (1-5) ________**
2. **What comorbidities do you currently have? (Multiple-choice)**

**○ Diabetes**

**○ Hypertension**

**○ Heart disease**

**○ None**

**○ Other**

1. **What is your marital status? (Single-choice)**

**○ Married**

**○ Unmarried**

**○ Divorced**

**○ Widowed**

1. **What is your employment status? (Single-choice)**

**○ Retired**

**○ Employed**

**○ Retired from public service**

**○ Unemployed or laid off**

**○ Student**

**○ Other**

1. **Who takes care of your daily needs when you are not hospitalized? (Single-choice)**

**○ Self-care**

**○ Spouse**

**○ Children**

**○ Other caregivers**

1. **What is your occupation? (If retired, please indicate your previous occupation) (Single-choice)**

**○ Professional and technical personnel (e.g., teacher, doctor, lawyer, accountant)**

**○ Service industry personnel (e.g., waiter, driver, sales clerk)**

**○ Freelancer (e.g., writer, artist, photographer, tour guide)**

**○ Worker (e.g., factory worker, construction worker, city sanitation worker)**

**○ Agriculture, livestock, fishery, mining**

**○ Public institution or government employee**

**○ Company staff**

**○ Other**

1. **What is your level of education? (Single-choice)**

**○ Below primary school**

**○ Primary school**

**○ Middle school**

**○ High school or vocational school**

**○ Associate degree or bachelor's degree**

**○ Master's degree or above**

1. **What is the level of education of your caregiver? (Skip this question if self-care) (Single-choice)**

**○ Below primary school**

**○ Primary school**

**○ Middle school**

**○ High school or vocational school**

**○ Associate degree or bachelor's degree**

**○ Master's degree or above**

1. **What is your household's average monthly disposable income per capita? (Single-choice)**

**○ Below 2,000 yuan**

**○ 2,000-4,000 yuan**

**○ 4,000-6,000 yuan**

**○ 6,000-8,000 yuan**

**○ 8,000-10,000 yuan**

**○ Above 10,000 yuan**

**Part 2: Motivation for Dietary Management in Chronic Kidney Disease**

| Survey Items | |
| --- | --- |
| **Measures** | **Items** |
| **Perceived vulnerability**  **(Five-point Scale:A.Not at all**  **B.Slightly applicable**  **C.Moderately applicable**  **D.Mostly applicable**  **E.Fully applicable)** | PV1: Loss of renal function is likely to happen to me if I do not follow a renal diet recommended by the clinician. |
|  | PV2: My chances of renal decline are very high if I keep eating salty, sweetened and oily food. |
|  | PV3: My chances of getting comorbidities like type 2 diabetes, hypertension, and cardiovascular disease are high if I do not follow the clinician’s advice. |
| **Perceived severity**  **(Five-point Scale：A.Strongly Disagree**  **B.Somewhat Disagree**  **C.Neutral**  **D.Somewhat Agree**  **E.Strongly Agree)** | PS1: Renal decline will have a negative impact on the everyday life and work of my family and caregivers. |
|  | PS2: Renal decline will have a negative impact on my own everyday life and work. |
|  | PS3: Renal decline will place financial burden on my family. |
|  | PS4: Renal decline may have a negative impact on my quality of life. |
|  | PS5: Renal decline will increase the chance of requiring frequent hospitalizations and hospital visits. |
|  | PS6: Renal decline will increase the chance of requiring dialysis or transplant treatment. |
| **Intrinsic rewards**  **(Five-point Scale：A.Strongly Disagree**  **B.Somewhat Disagree**  **C.Neutral**  **D.Somewhat Agree**  **E.Strongly Agree)** | IR1: Some food restricted for kidney health is really tasty and delightful. |
|  | IR2: I think an unrestricted diet is better for overall nutrition and health. |
|  | IR3: I think an unrestricted diet makes me feel more relaxed. |
|  | IR4: I think an unrestricted diet makes me enjoy life more. |
|  | IR5: I think an unrestricted diet makes me feel better about myself. |
| **Extrinsic rewards**  **(Five-point Scale:A.Not at all**  **B.Slightly applicable**  **C.Moderately applicable**  **D.Mostly applicable**  **E.Fully applicable)** | ER1: My family prefer I do not restrict intake of certain food or food groups because of kidney condition. |
|  | ER2: My family prefer that I continue to eat organ meats and highly nutritious seafood as much as I can to avoid malnutrition. |
|  | ER3: My family think an unrestricted diet is better for overall nutrition and health. |
|  | ER4: My family think it is easier to prepare food if I maintain an unrestricted diet. |
| **Response efficacy**  **(Five-point Scale：A.Strongly Disagree**  **B.Somewhat Disagree**  **C.Neutral**  **D.Somewhat Agree**  **E.Strongly Agree)** | RE1: Following scientific dietary guidance can help improve my nutrition and health. |
|  | RE2: Dialysis may be delayed or avoided by following a renal diet. |
|  | RE3: Following a healthy diet can slow the impairment of kidney function. |
|  | RE4: Following a healthy diet can curb the development and progression of diabetes, hypertension and other comorbidities. |
|  | RE5: Keeping a healthy diet will improve my quality of life. |
| **Self-efficacy**  **(Five-point Scale:A.Not at all**  **B.Slightly applicable**  **C.Moderately applicable**  **D.Mostly applicable**  **E.Fully applicable)** | SE1: I am confident in keeping a healthy diet for the sake of improving my kidney condition. |
|  | SE2: I have the knowledge and skills required for getting the right type and amount of food suitable for my condition. |
|  | SE3: I know what to do with situations that interfere with my dietary plan (e.g. social dining with people who order mostly unhealthy food). |
|  | SE4: I know what to do with situations that interfere with my dietary plan (e.g. being busy and having to order takeout). |
|  | SE5: I know what to do with situations that interfere with my dietary plan (e.g. traveling). |
| **Response cost**  **(Five-point Scale：A.Strongly Disagree**  **B.Somewhat Disagree**  **C.Neutral**  **D.Somewhat Agree**  **E.Strongly Agree)** | RC1: Following a renal diet requires more efforts in preparing food. |
|  | RC2: Following a renal diet hinders my social life. |
|  | RC3: Following a renal diet is really challenging in practice. |
|  | RC4: Following a renal diet is stressful. |
|  | RC5: Following a renal diet costs me more than I gain. |
| **Dietary management intention**  **(Five-point Scale：A.Strongly Disagree**  **B.Somewhat Disagree**  **C.Neutral**  **D.Somewhat Agree**  **E.Strongly Agree)** | DMI1: I intend to study information on dietary management for kidney disease after hospital discharge. |
|  | DMI2: I intend to reduce salt intake after hospital discharge. |
|  | DMI3: I intend to follow a renal diet back home after hospital discharge. |
|  | DMI4: I intend to follow a healthy diet after hospital discharge. |
|  | DMI5: I intend to eat healthily on all occasions. |
| Note: IR1, IR5, and RC1 were removed from based on results from confirmatory factor analysis. | |

**慢性肾病自我管理行为和动机问卷**

尊敬的病友，我们是上海中医药大学附属龙华医院与上海交通大学合作的健康传播研究团队，在对慢性肾病患者疾病自我管理行为、动机和认知情况进行调查，为慢性肾病患者疾病注意事项和健康传播策略设计提供参考依据。所有收集的信息会进行匿名化和保密处理，且仅用于公共卫生和健康传播课题研究，对治疗无影响，请您根据真实情况填写即可。非常感谢您的配合！

**第一部分：基本信息**

1、 ID （填空题）

2、您的出生年月日是 （填空题）

3、您的性别是 （单选题）

○男 ○女

4、请填写以下内容 (填空题)

您的身高是 (厘米，cm)

您的体重是 (公斤，kg)

您被确诊为慢性肾病几个月（年）了？

目前属于慢性肾病第几期？（1-5）

1. 您目前出现的合并症有（多选题）

○糖尿病 ○高血压 ○心脏病 ○无 ○其他

1. 您的婚姻状况是 （单选题）

○已婚 ○未婚 ○离异 ○丧偶

1. 您的工作状况是 （单选题）

○退休 ○在职 ○离休 ○下岗或无业 ○学生 ○其他

1. 非住院期间谁在照顾您日常起居？ （单选题）

○自我照顾 ○配偶照顾 ○子女照顾 ○其他人照顾

1. 您的职业是？（如为离退休，请填写离退休之前的职业） （单选题）

○专业技术人士（如教师/医生/律师/会计师等） ○服务业人员（餐饮服务员/司机/售货员等）

○自由职业者（如作家/艺术家/摄影师/导游等） ○工人（工厂工人/建筑工人/城市环卫工人）

○农牧渔矿业 ○事业单位/公务员/政府工作人员 ○公司职员 ○其他

10、您的文化程度是？ （单选题）

○小学以下 ○小学 ○初中 ○高中或者中专 ○大专或者本科 ○硕士或者以上

1. 照顾者的文化程度**（自我照顾者跳过此题）** （单选题）

○小学以下 ○小学 ○初中 ○高中或者中专 ○大专或者本科 ○硕士或者以上

1. 您的家庭人均每月可支配收入是？ （单选题）

○2000 元以下 ○2000-4000 元 ○4000-6000 元

○6000 元-8000 元 ○8000-10000 元 ○10000 元以上

**第二部分：慢性肾病饮食管理动机**

14、（打钩题）

|  | 完全不可能 | 比较不可能 | 有点可能 | 比较可能 | 非常可能 |
| --- | --- | --- | --- | --- | --- |
| 作为慢性肾病患者，若不按照肾病原则进行饮食管理就会加速肾功能恶化 |  |  |  |  |  |
| 作为慢性肾病患者，若继续食用高盐、高糖、高油食物，就会加速肾功能恶化 |  |  |  |  |  |
| 作为慢性肾病患者，若不按照医生的建议进行管理，就会增加患上2型糖尿病、高血压和心血管疾病等并发症的风险 |  |  |  |  |  |

15、 （打钩题）

|  | 完全不同意 | 比较不同意 | 中立 | 比较同意 | 完全同意 |
| --- | --- | --- | --- | --- | --- |
| 如果我的病情恶化（肾功能下降），会对家人和照顾者的日常生活和工作产生负面影响 |  |  |  |  |  |
| 如果我的病情恶化（肾功能下降），会对我自己的日常生活和工作产生负面影响 |  |  |  |  |  |
| 如果我的病情恶化（肾功能下降），会给我的家庭带来经济负担 |  |  |  |  |  |
| 如果我的病情恶化（肾功能下降），会对我的生活质量产生负面影响 |  |  |  |  |  |
| 如果我的病情恶化（肾功能下降），会增加我需要频繁住院和就诊的机会 |  |  |  |  |  |
| 如果我的病情恶化（肾功能下降），会增加我需要透析或移植治疗的可能性 |  |  |  |  |  |

16、请选择符合您实际情况的选项 （打钩题）

|  | 完全不同意 | 比较不同意 | 中立 | 比较同意 | 完全同意 |
| --- | --- | --- | --- | --- | --- |
| 为肾脏健康而限制的食物非常美味可口 |  |  |  |  |  |
| 我觉得不受限制的饮食更利于全面营养和健康 |  |  |  |  |  |
| 我觉得不受限制的饮食让我感到更轻松 |  |  |  |  |  |
| 我觉得不受限制的饮食让我感到生活更有乐趣 |  |  |  |  |  |
| 不受限制的饮食让自己感觉更舒服 |  |  |  |  |  |

17、我家人 （打钩题）

|  | 完全不符合 | 小部分符合 | 一半符合 | 大部分符合 | 完全符合 |
| --- | --- | --- | --- | --- | --- |
| 我家人希望我不要针对肾病刻意限制某些食物种类的摄入 |  |  |  |  |  |
| 我家人希望我尽可能多摄入动物内脏和富含营养的海鲜之类的食物以保证充足营养 |  |  |  |  |  |
| 我家人认为不受限制的饮食更利于我全面营养和健康 |  |  |  |  |  |
| 我家人认为不受限制的饮食更便于准备食物 |  |  |  |  |  |

18、请为您对以下陈述的认同度打分，没有对错之分 （打钩题）

|  | 完全不同意 | 比较不同意 | 中立 | 比较同意 | 完全同意 |
| --- | --- | --- | --- | --- | --- |
| 遵循科学的饮食指导可以帮助改善我的营养和健康 |  |  |  |  |  |
| 遵循肾脏饮食可能会延缓或避免透析的需要 |  |  |  |  |  |
| 遵循健康饮食可以减缓肾功能的损害 |  |  |  |  |  |
| 遵循健康饮食可以抑制糖尿病、高血压及其他并发症的发展和进展 |  |  |  |  |  |
| 保持健康饮食将改善我的生活质量 |  |  |  |  |  |

19、请选择符合您实际情况的选项 （打钩题）

|  | 完全不符合 | 小部分符合 | 一半符合 | 大部分符合 | 完全符合 |
| --- | --- | --- | --- | --- | --- |
| 我有信心通过保持健康饮食来改善我的肾脏状况 |  |  |  |  |  |
| 我具备选择适合我情况的食物种类和数量所需的知识和技能 |  |  |  |  |  |
| 我知道如何应对干扰我饮食计划的情况（如与家人或者朋友共同就餐时他人不健康饮食）， |  |  |  |  |  |
| 我知道如何应对干扰我饮食计划的情况（如工作很忙，而外出就餐或者外卖）， |  |  |  |  |  |
| 我知道如何应对干扰我饮食计划的情况（如离家去外地出差或远途旅行）， |  |  |  |  |  |

20、请选择符合您实际情况的选项 （打钩题）

|  | 完全不同意 | 比较不同意 | 中立 | 比较同意 | 完全同意 |
| --- | --- | --- | --- | --- | --- |
| 遵循肾脏饮食需要在准备食物方面投入更多精力 |  |  |  |  |  |
| 遵循肾脏饮食妨碍了我的社交生活。 |  |  |  |  |  |
| 实践中遵循肾脏饮食确实具有挑战性 |  |  |  |  |  |
| 遵循肾脏饮食让我倍感压力 |  |  |  |  |  |
| 遵循肾脏饮食的成本大于收益 |  |  |  |  |  |

21、请选择符合您实际情况的选项，我将 （打钩题）

|  | 完全不同意 | 比较不同意 | 中立 | 比较同意 | 完全同意 |
| --- | --- | --- | --- | --- | --- |
| 我打算出院后学习肾脏疾病的饮食管理信息 |  |  |  |  |  |
| 我打算出院后减少盐的摄入 |  |  |  |  |  |
| 我打算出院后在家遵循肾脏饮食 |  |  |  |  |  |
| 我打算出院后遵循健康饮食 |  |  |  |  |  |
| 我打算在所有情况下都保持健康饮食 |  |  |  |  |  |
