# Supplementary material for: Predicting dietary management intention of patients with chronic kidney disease using protection motivation theory
Source: PLoS One. 2025 Mar 18;20(3):e0320340. doi: 10.1371/journal.pone.0320340 (PMC11957771; doi:10.1371/journal.pone.0320340)
Supplement: Attachment — Submitted filename: comments for Predicting DMI of Patients with CKD Using PMT.doc [file pone.0320340.s005.doc]

Reviewers comments for Predicting Dietary Management Intention of Patients with Chronic Kidney Disease Using Protection Motivation Theory

1) Keep the Dietary Management Intentions- consistent

Not Intention in one place and intentions in other places

2) Introduction:

Chronic kidney disease (CKD) is a pathological condition in which a gradual loss

of kidney function occurs over five stages classified by estimated glomerular filtration

rate [1]. This is a clinical classification and classified into 5 stages- needs correction

As patients in early stages of CKD might have few signs or symptoms, they

may feel reluctant to seek and follow medical advice on managing the condition.

This statement makes more sense if it says less instead of few.

Recommendations to do this study on an outpatient basis as well.
